# Supplementary figures and images for: Biomechanical, Anthropometric and Psychological Determinants of Barbell Bench Press Strength
Source: Sports (Basel). 2022 Dec 5;10(12):199. doi: 10.3390/sports10120199 (PMC9785143; doi:10.3390/sports10120199)

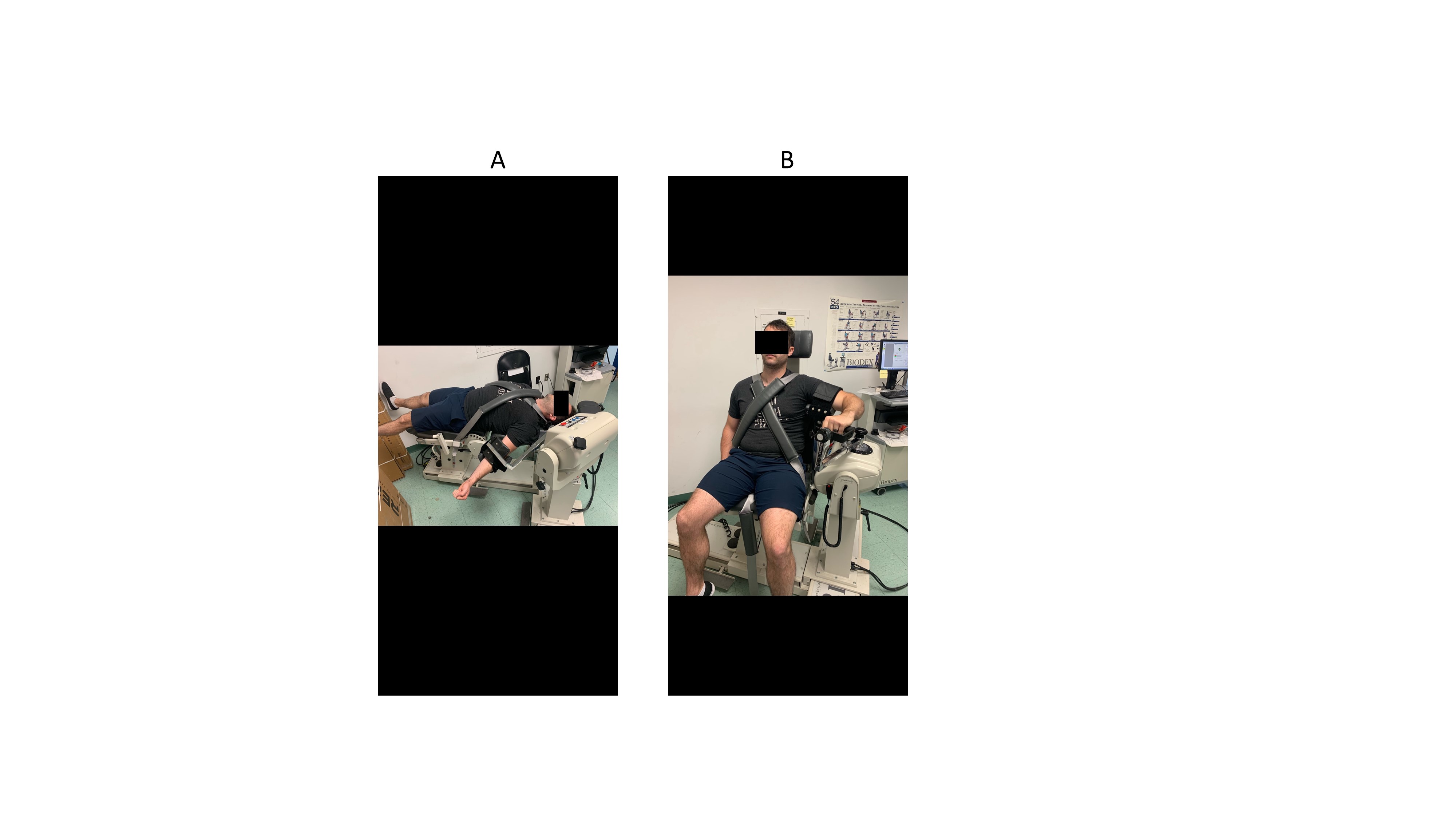

Supplement: Supplementary file 1 [file sports-10-00199-s001.zip › sports-2072423-supplementary.jpg]
